# Design optimization of a linkage-based 2-DOF wheel mechanism for stable step climbing

**DOI:** 10.1038/s41598-022-21410-1

**Published:** 2022-10-07

**Authors:** Jeeho Won, Sijun Ryu, Sangkyun Kim, Kwan Yeong Yoo, Hwa Soo Kim, Taewon Seo

**Affiliations:** 1 Department of Mechanical Convergence Engineering, Hanyang University, Seoul, 04763 Republic of Korea; 2 Department of Mechanical Systems Engineering, Kyonggi University, Suwon, 16227 Republic of Korea

**Keywords:** Engineering, Mechanical engineering

## Abstract

This paper presents the design optimization of a linkage-based wheel mechanism with two degrees of freedom, for stable step climbing. The mechanism has seven rotational joints and one prismatic joint. Kinematic and dynamic analyses of the mechanism were performed. The design was optimized in terms of linkage length and architecture to better manipulate the mechanism in its workspace, which was defined here by the targeted step size, as well as to ensure stability while climbing stairs. Optimization by genetic algorithm was performed using MATLAB. The optimized mechanism exhibited enhanced torque transmission from the input torque to the exerted for at the lobe of the wheel. Compliance control of the transformation will be addressed in the future.

## Introduction

Wheels are very effective for locomotion on flat surfaces but are not suitable for climbing. Most modern vehicles and mobile robots use wheels for locomotion on horizontal terrains. However, wheels are disadvantageous in the case of steps, requiring steps smaller than the wheels’ radii, to overcome the rotation torque. However, steps are ubiquitous in human environments, making it difficult for mobile robots to perform well in these environments.

Several attempts have been made to design wheels that would allow climbing such steps. Robots such as ASGUARD use fixed spokes to overcome stepped obstacles^1,2^. RHex, Loper, and IONS use wheel-legged mechanisms, with wheel rims as legs, for climbing steps^3–5^. While these robots are able to overcome various obstacles, including steps, their fixed design implies suboptimal performance at different step sizes. This is important, because stair step sizes appear to vary widely, i.e., they are likely to be different for indoors/outdoors, adult/minor use, and commercial/residential settings^6–9^. A previous study proposed a curved spoke wheel optimized for stair climbing, suggesting an optimal trajectory for wheel-legged robots^10^. The proposed curved spoke wheel also has a fixed design, but the design parameters can be adjusted for different stair types. Step-size targeted designs can potentially solve this problem; however, fixed designs tend to destabilize platforms on flat surfaces, compared to regular wheels.

Instead of fixed-wheel designs, some studies have focused on transformable wheels that can benefit from both fixed climbable wheels and regular wheels. Many robots use transformable wheel-leg designs with one degree of freedom (1-DOF), for climbing steps^11–17^. Krys et al. also suggested transformable wheels for climbing steps^18^. Such 1-DOF transformable wheel mechanisms can change regular-shaped wheels into climbable wheels; however, these designs remain inflexible with respect to various step sizes.

To adapt to different step sizes and maintain stability on flat surfaces, a transformable wheeled robot with two degrees of freedom (2-DOF), named STEP, has been proposed^19^. This mechanism can adjust its diameter and the rotation of its three parted rims, for climbing different-size steps. However, the mechanism is extremely bulky. Subsequent research was conducted to make the mechanism more compact and modular^20^. The proposed modular design has two rotational inputs with seven links containing six rotational joints and one prismatic joint, thus creating the 2-DOF mechanism. The linkage length of the modular wheel is determined using the largest target step size. Although the mechanism’s manipulability has been analyzed, the index has not been improved.

**Figure 1:**
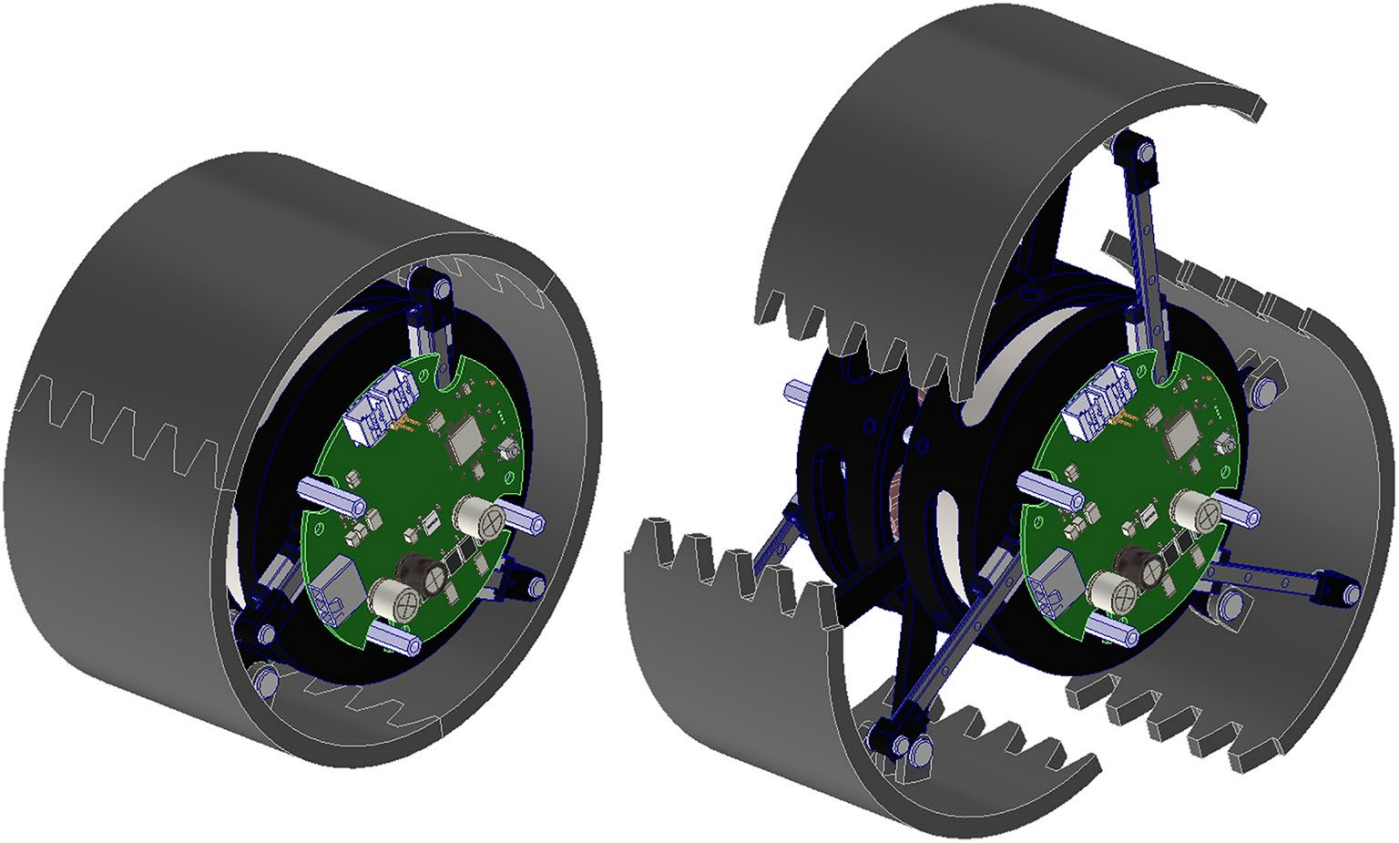
CAD model of the transformable wheel. The three lobes that form the rim of the wheel have two DOFs generated by two BLDC motors inside the wheel, which can transform into leg shapes capable of climbing stairs.

The modular design of transformable wheels suffers from some imperfections. While the wheels’ kinematic analysis was performed to derive the mechanism’s Jacobian, dynamic analysis was not performed on the initially proposed design. The correlation between the input torque produced by the transformation motors and the output force of the three separate rims remains unknown. The linkage lengths were determined to be the largest target step, as proposed by the STEP development study^20^. However, the linkage lengths were not determined in calculations; only the mechanism’s functionality was determined. The design also accounts the symmetry in the linkage designs targeted for easy manufacture. However, the design of the wheel is innately asymmetrical due to the inevitable location of the LM guide, which cannot be located in the center of the rim rotation for avoiding interference with each other. In addition, previous research did not consider the fact that the mechanism consists of three identical 2-DOF planar parallel 7-bar linkage manipulators with two rotational inputs combined to form the shape of a wheel, as shown in Fig. 1. Normally, a 7-bar planar linkage manipulator would create three DOFs. One end of the end-effector was constrained to a prismatic joint, yielding two DOFs for this mechanism.

In previous studies concerning parallel mechanisms, workspace and stiffness optimization has been performed. Liu and Wang used performance indices such as the global conditioning index (GCI), the global velocity index (GVI), the global payload index (GPI), and the global stiffness index (GSI)^21^. The maximal inscribed circle and maximal inscribed workspace were introduced for kinematic optimization. Some redundantly actuated parallel mechanisms have also been considered; for example, Shin optimized the antagonistic stiffness of a redundantly actuated parallel manipulator^22^. The transformable wheel mechanism is also a parallel mechanism, however its performance solely focused on the functionality which is the maximum height of the climbable step.

In this study, the kinematics and workspace, along with the dynamics of the transformable wheel mechanism required for the 2-DOF wheel for stable step climbing, are derived in “Kinematics of the transformable wheel” section. In “Optimization of linkage lengths” section, linkage designs are optimized with respect to the stability or the maximal torque usage of the transformation motors within the derived workspace along with scoring of the torque performance of the two motor inputs. The conclusions of the work and the dynamics optimization prospects are discussed in “Results and analysis” section.

## Kinematics of the transformable wheel

### 2-DOF transformable wheel

Wheel kinematics is crucial for optimization. The input-DOF relationship must be clarified before using the Jacobian for investigating the GCI. A previous work discussed the kinematics of the transforming wheel mechanism; however, it was not successful in concluding with successive analytic calculations. Here, before the kinematic calculations, the kinematic setup of the mechanism was examined.

**Figure 2:**
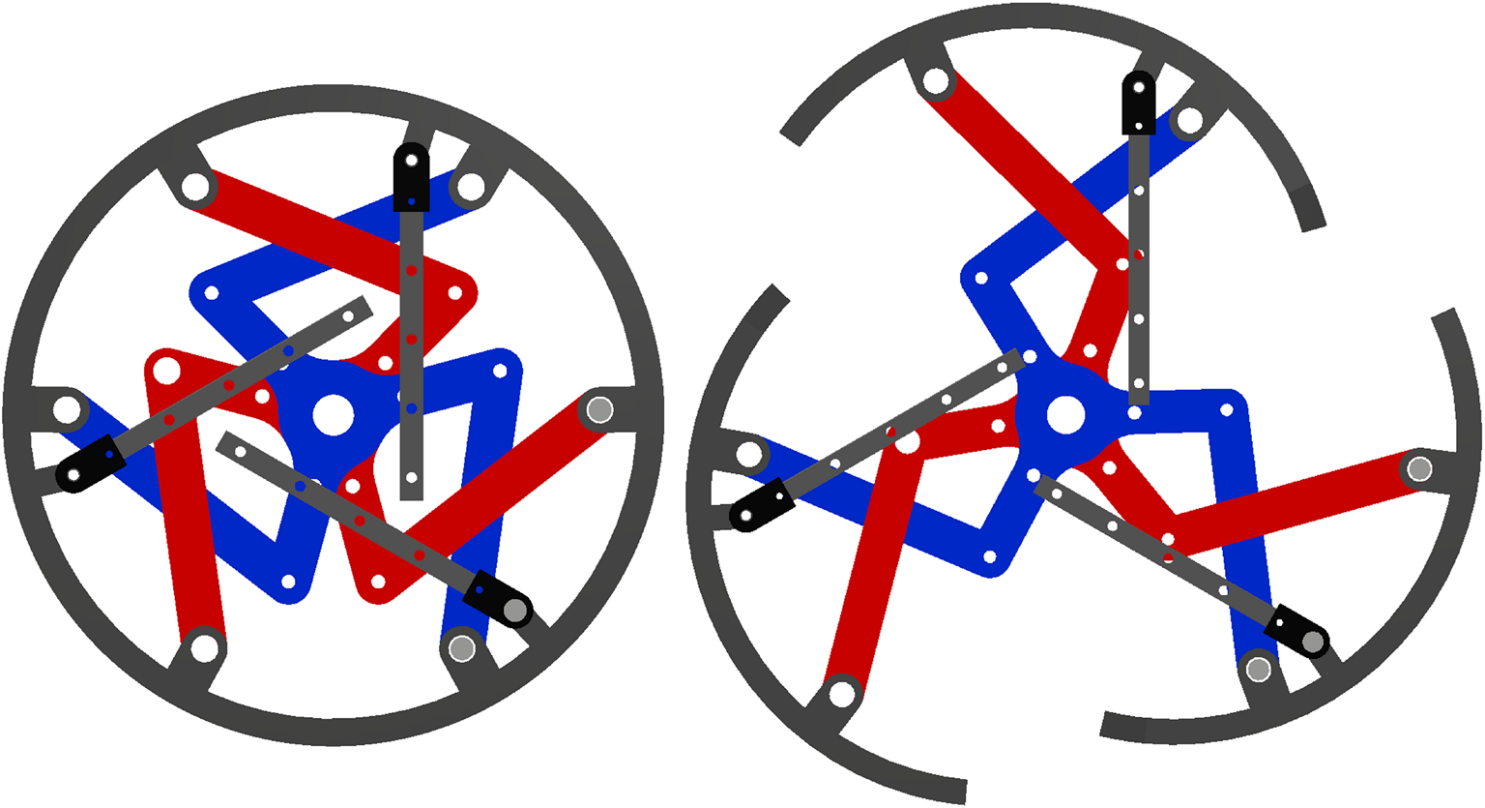
CAD model with only the linkages. The center triangle linkages (blue and red) are connected to a BLDC motor to create motion and torque. Each lobe consists of identical kinematic configurations, which makes the analysis of one lobe equal to that of the entire wheel.

The transformable wheel was composed of three identical linkage connections for each of the three lobes, as shown in Fig. 2. The two center linkages were connected to a brushless DC (BLDC) motor to create motion and torque. The center linkages were identical in the $120^\circ$ direction; thus, every kinetic movement of a lobe was identical to that of the other lobes. Owing to this similarity, it was possible to analyze the kinematics of only one lobe, and then rotate and duplicate for the kinematic analysis of the entire wheel. Each lobe consisted of a 7-bar linkage with one prismatic joint. Kinematic and dynamic analyses were based on one lobe in this study.

**Figure 3:**
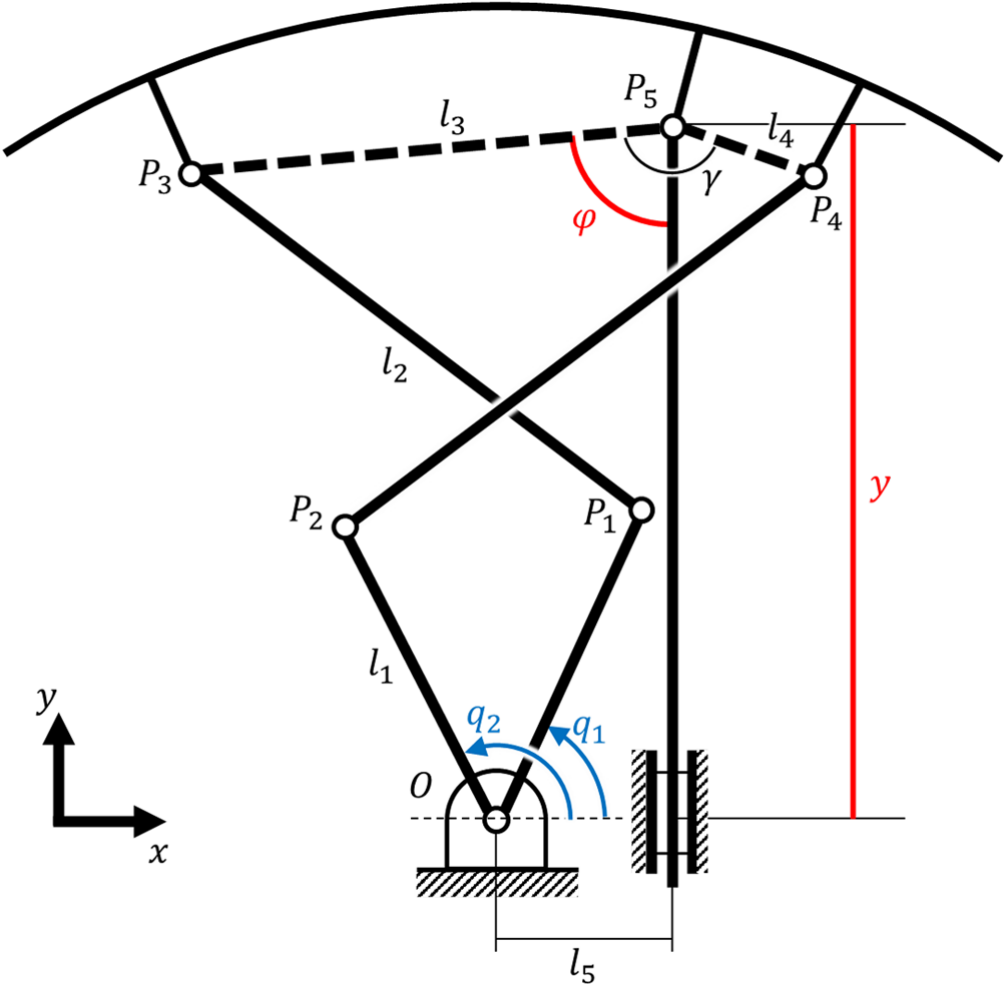
Kinematic diagram of the 2-DOF 7-bar linkage. Two inputs $q_1$ and $q_2$ yield two DOFs in $\varphi$ and $y$.

A kinematic diagram of the 7-bar transformable mechanism is shown in Fig. 3. Six bars, including the rim of the wheel, were connected to revolute joints. This configuration resulted in three DOFs, where the rim rotated along the reference frame. A prismatic joint bar was connected to a rotary joint at one point of the wheel rim, while the other end was affixed at 90 degrees to the reference frame. This prismatic joint created a constraint on the mechanism, to prevent the rim from rotating relative to the reference. Thus, the rim had two DOFs, where it rotated at $P_5$ for $\varphi$ and translated in the $y$ direction owing to the $90^\circ$ constraint for length $y$. A similar configuration has been studied with two parallel revolute actuators for seven rotation joints and one prismatic joint, thus creating a 2-DOF system^23^. This paper presents analytic kinematics for a configuration identical to the kinematics of a transformable wheel. The following analysis was based on this research.

### Forward kinematics

A kinematic diagram of the mechanism is shown in Fig. 3, where $q_1,q_2$ are the two parallel inputs of the mechanism. The lengths of the links $l_1 , l_2, l_3, l_4$ are given, and the angles $\gamma$ as points $P_3, P_4, P_5$ are geometrically constrained to the rim. Parameter $l_5$ is the length between the actuator center and the prismatic linkage. The coordinates of points $P_1$ and $P_2$ can be defined using $q_1, q_2$, where points $P_3, P_4, P_5$ can be defined using the two unknowns $\varphi$ and $y$.

1 $$ P_{1} :(l_{1} \cos q_{1} ,l_{1} \sin q_{1} ) $$

2 $$ P_{2} :(l_{1} \cos q_{2} ,l_{1} \sin q_{2} ) $$

3 $$ P_{3} :(l_{5}  - l_{3} \sin \varphi ,y - l_{3} \cos \varphi ) $$

4 $$\begin{aligned}{}&\quad P_4 : (l_5 - l_4\sin {(\gamma - \varphi )},y - l_4\cos {(\gamma - \varphi )}) \end{aligned}$$

From the geometry of the mechanism, the vector lengths $|\overline{P_1P_3}|$ and $|\overline{P_2P_4}|$ can be expressed using the following two equations:

5 $$ (l_{1} \cos q_{1}  - (l_{5}  - l_{3} \sin \varphi ))^{2}  + (l_{1} \sin q_{1}  - (y - l_{3} \cos \varphi ))^{2}  - l_{2}^{2}  = 0 $$

6 $$ (l_{1} \cos q_{2}  - (l_{5}  - l_{4} \sin (\gamma  - \varphi )))^{2}  + (l_{1} \sin q_{2}  - (y - l_{4} \cos (\gamma  - \varphi ))^{2}  - l_{2}^{2}  = 0{\text{ }} $$

For $\varphi$, it can be substituted as follows:

7 $$\begin{aligned} \cos {\varphi } = \frac{1-t^2}{1+t^2} \end{aligned}$$

where $t= \tan {\varphi /2}$, $\varphi = 2t/1+t^2$. From Eqs. (5), (6), and (7), we obtain two second-order polynomial equations in *t* as follows:

8 $$\begin{aligned} (A_i - B_i)t^2 + 2C_it +(A_i+B_i) = 0 \end{aligned}$$

where

$$\begin{aligned} A_1&= l_1^2 + l_5^2 -l_2^2+l_3^2+ y^2 - 2l_1l_5\cos {q_1}-2l_1y\sin {q_1} \\ B_1&= 2l_1l_3\sin {q_1}-2l_3y \\ C_1&= 2l_1l_3\cos {q_1}-2l_3l_5 \\ A_2&= l_1^2 + l_5^2 - l_2^2 + l_4^2+ y^2-2l_1l_5\cos {q_2} - 2l_1y\sin {q_2} \\ B_2&= 2l_4l_5\sin {\gamma }+2l_1l_4\cos {\gamma }\sin {q_2} -2l_1l_4\cos {q_2}\sin {\gamma } - 2l_4y\cos {\gamma } \\ C_2&= 2l_1l_4\cos {q_2}\cos {\gamma }+2l_1l_4\sin {q_2}\sin {\gamma }-2l_3l_5\cos {\gamma }-2l_4y\sin {\gamma } \end{aligned}$$

For Eq. (8) to have a common solution, the following should be satisfied, where

9 $$\begin{aligned} \begin{pmatrix} A_1 - B_1 &{} \quad 2C_1 &{} \quad A_1+B_1 \\ A_2 - B_2 &{} \quad 2C_2 &{} \quad A_2+B_2 \end{pmatrix} \begin{pmatrix} t^2 \\ t \\ 1 \end{pmatrix} = 0 \end{aligned}$$

However, as the matrix is not square, it is difficult to solve for its determinant. To overcome this, *t* is multiplied by Eq. (8) to yield a third-order equation, where the former second-order equation is thought to have 0 as its $t^3$ polynomial. Consequently, the determinant of the polynomial equation must be zero, for satisfying the above equations.

10 $$ \left| {\begin{array}{*{20}c}    0 & {A_{1}  - B_{1} } & {2C_{1} } & {A_{1}  + B_{1} }  \\    {A_{1}  - B_{1} } & {2C_{1} } & {A_{1}  + B_{1} } & 0  \\    0 & {A_{2}  - B_{2} } & {2C_{2} } & {A_{2}  + B_{2} }  \\    {A_{2}  - B_{2} } & {2C_{2} } & {A_{2}  + B_{2} } & 0  \\   \end{array} } \right| = 0{\text{ }} $$

Because $A_i,B_i,C_i$ is expressed with *y*, by expanding Eq. (10), we obtain a sixth-order polynomial equation in *y* as follows:

11 $$\begin{aligned} (A_1B_2 - A_2B_1)^2+(A_2C_1-A_1C_2)^2 - (B_2C_1-B_1C_2)^2 = 0 \end{aligned}$$

By solving for *y*, Eq. (8) can be solved for *t* using a quadratic formula. Only one solution for *y* out of the six satisfies Eqs. (5) and (6), where *t* is also determined.

### Inverse kinematics

Similar to forward kinematics, inverse kinematics can be determined by substituting a trigonometric term with a linear term. The two required outputs $\varphi$ and *y* can be described by two unknown inputs, $q_1$ and $q_2$. In Eqs. (5) and (6), the input variables can be substituted as follows:

12 $$\begin{aligned} \cos q_i = \frac{1-s_i^2}{1+s_i^2} \end{aligned}$$

where $s_i = \tan {(q_i/2)}$ , for $i = 1,2$, to obtain second-order polynomials in $s_i$ as

13 $$\begin{aligned} (F_i-D_i)s_i^2+2E_is_i+(F_i+D_i) = 0 \end{aligned}$$

where

$$\begin{aligned} D_1&= 2l_1(l_5 - l_3\sin {\phi }) \\ E_1&= 2l_1(l_3\cos {\phi } - y) \\ F_1&= -\frac{(D_1^2+E_1^2)}{(4l_1^2)} + l_2^2-l_1^2 \\ D_2&= 2l_1(l_5 + l_4\sin {(\phi - \gamma )} \\ E_2&= 2l_1(l_4\cos {(\phi - \gamma )} - y) \\ F_2&= -\frac{(D_2^2+E_2^2)}{(4l_1^2)} + l_2^2-l_1^2 \end{aligned}$$

For a given $(y,\phi )$, $s_i$ can be solved by using a quadratic formula. Therefore, inverse kinematics can be obtained using

14 $$\begin{aligned} s_i = {\left\{ \begin{array}{ll} 2 \tan ^{-1}{ \left( \frac{-E_i \pm \sqrt{D_i^2+E_i^2-F_i^2}}{F_i - D_i} \right) } &{} \quad \text {when} \left( F_i-D_i\right) \ne 0 \\ -\frac{F_i+D_i}{2E_i} &{} \quad \text {when} \left( F_i-D_i\right) = 0 \end{array}\right. } \end{aligned}$$

As a result, the required input angles of the transformed motors for a given posture can be obtained.

### Jacobian, workspace, and manipulability

Equation (11) can be rewritten as a sixth-degree polynomial equation with respect to *y* as follows:

15 $$\begin{aligned} f(y) = 0 \end{aligned}$$

where *f* is a polynomial expression represented by $[q_1,q_2]$ for *y*. Because $[q_1,q_2]$ is also time-dependent, generalized inputs $[\dot{q_i},\ddot{q_i}]$ exist. By differentiating Eq. (15) with respect to time, we obtain

16 $$\begin{aligned} \frac{\partial f}{\partial q_1}\dot{q_1} + \frac{\partial f}{\partial q_2}\dot{q_2} + \frac{\partial f}{\partial y}{\dot{y}} = 0 \end{aligned}$$

In this manner, ${\dot{y}}$ can be expressed with $\dot{q_i}$. In forward kinematics, by using the quadratic formula for the quadratic equation given in Eq. (8), $t_i$ can be expressed using $[q_1,q_2,y]$ with respect to time.

17 $$\begin{aligned}&t_i = \frac{-C_i \pm \sqrt{C_i^2 - 4(A_i-B_i)(A_i+B_i)}}{2(A_i-B_i)} = g(q_1,q_2,y) \end{aligned}$$

18 $$\begin{aligned}&\quad \therefore \dot{t_i} = \frac{\partial g}{\partial q_1}\dot{q_1} + \frac{\partial g}{\partial q_2}\dot{q_2} + \frac{\partial g}{\partial y}{\dot{y}} = 0 \end{aligned}$$

As ${\dot{y}}$ can be expressed with $\dot{q_i}$, $t_i$ can be expressed using $\dot{q_i}$. Consequently, from the definition of $t_i$, the output degree of freedom ${\dot{\phi }}$ is obtained. Because ${\dot{y}}$ and ${\dot{\phi }}$ are both expressed by $\dot{q_1} , \dot{q_2}$, the Jacobian of the mechanism can be defined as

19 $$\begin{aligned} {\dot{\Phi }} = J{\dot{q}} \end{aligned}$$

where *J* is a $2 \times 2$ square matrix with ${\dot{\Phi }} = [y \ \phi ]^T$ and $q = [q_1 \ q_2]^T$.

The geometry of the motor armature of the wheel limits the workspace of the two actuators. Therefore, it is important to determine the optimal condition in which the wheel operates within a specific region. The workspaces of the two actuators are symmetrical based on the Y-axis, as shown in Fig. 3. The initial conditions of the existing model of the transformable wheel are given in Table 1. Despite their small region, three lobes create a sufficiently wide coverage to climb the target height steps.

**Table 1:** Initial conditions of the existing model of the transformable wheel. The two input angles $q_1$ and $q_2$ have about $40^\circ$ of workspace towards the center of their initial positions.

Name	$q_1$	$q_2$	$l_1$	$l_2$	$l_3$	$l_4$	$\gamma$
value	$45.105^\circ$	$134.895^\circ$	55 mm	75.49 mm	52.61 mm	19.68 mm	$147.42^\circ$

The manipulability of the mechanism inside the limited area can be calculated. The manipulability of the mechanism can be described using the Jacobian method presented by Salisbury and Creig^24^.

20 $$\begin{aligned} w = \frac{1}{cond\left( J\right) } \end{aligned}$$

**Figure 4:**
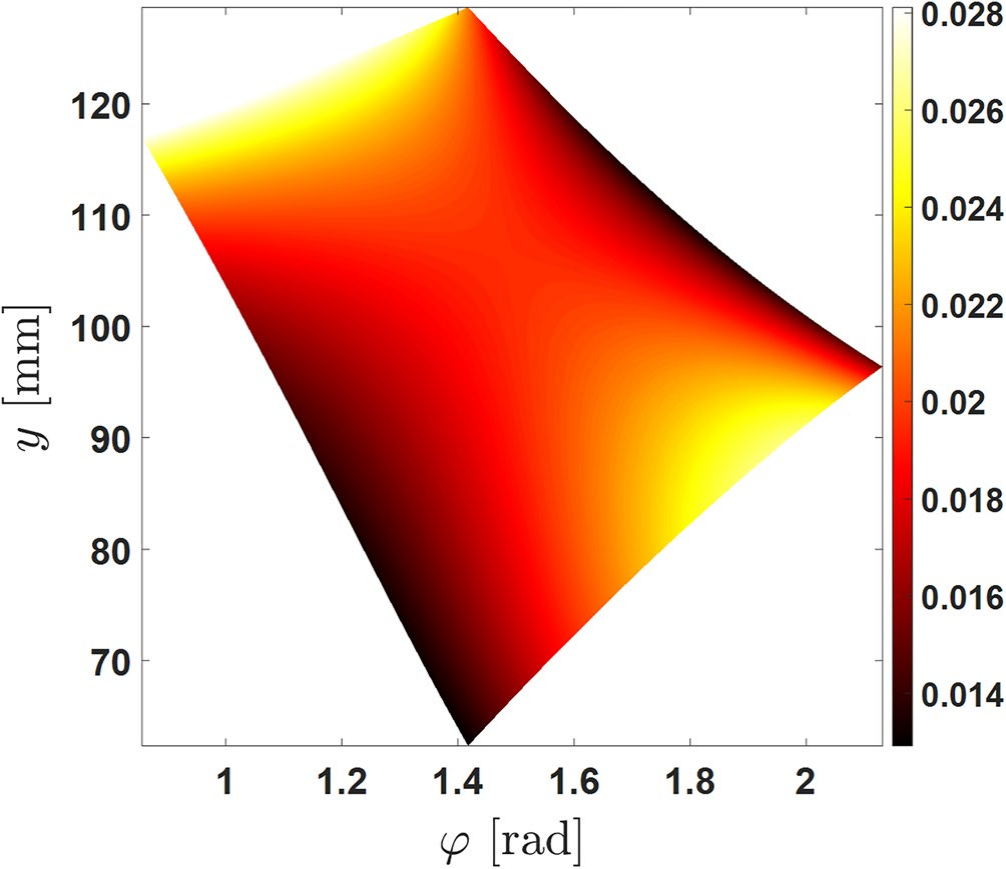
Manipulability of the mechanism in the given workspace. The workspace of the 2 DOF mechanism can be visualized with this graph. The manipulator workspace is configured as described in Table 1.

The manipulability of the mechanism inside the presented workspace is as in Fig. 4.

### Dynamics

It is possible to evaluate the dynamic characteristics of a device using a numerical approach. However, because this device is expected to be controlled in various ways, such as compliance control, it is crucial for the mechanism to have a dynamic model. With dynamic analysis, it is possible to determine how much force is exerted on a lobe by the two motor torques during the wheel’s transformation. Although the mechanism is highly nonlinear, it is possible to derive analytic equations using kinematics. From Eq. (11), the solution of the equation cannot be directly expressed with *q*. However, because this equation is for both *y* and *q*, the derivation of the two variables can be accessed. The coefficients’ expressions in this equation are quite cumbersome, making the derivatives extremely complex. However, the expressions obtained using these complex equations can be calculated faster via simulations. To illustrate the approach, the Euler–Lagrange equation was derived. To focus on the force exerted by the transformation motors, the wheel axes were set tangentially to the ground, neglecting the gravity during the transformation process. It is possible to consider gravity as it plays a crucial role in terms of lifting the wheel during the transformation. However, if gravity is added in the dynamics, the results would not be subjected to the input torques of the wheel, which is the focus of the optimization which will be addressed later in the study. Therefore, the *V* product of the Lagrangian was neglected in this calculation. The right-hand side of the Euler-Lagrange equation represents the input torque, which is the input torque owing to the transformation motor.

21 $$\begin{aligned} \begin{aligned} L = T - V , V = 0 \\ \frac{d}{dt}\left( \frac{\partial L}{\partial {\dot{q}}}\right) - \frac{\partial L}{\partial q} = \tau \end{aligned} \end{aligned}$$

The kinetic energy of the mechanism consists of the rotation and translation components of different bodies, where the corresponding velocities can be derived from the Jacobian. Because there are two center rotation linkages, three sets of two connecting linkages, and three lobes in the mechanism, the kinetic energy of each body is considered.

22 $$\begin{aligned} T = \frac{1}{2}n{\dot{Q}}^TM{\dot{Q}} \end{aligned}$$

where *M* is the mass term, ${\dot{Q}}$ is the velocity term, *n* indexes a specific body, and $\psi _1$ and $\psi _2$ are the angular velocities of the two linkages $\overline{P_1P_3}$ and $\overline{P_2P_4}$, respectively.

$$\begin{aligned} M&= diag(m_{1_{q_1}},J_{1_{q_1}},m_{1_{q_2}},J_{1_{q_2}},m_{2_{q_1}},J_{2_{q_1}},m_{2_{q_2}},J_{2_{q_2}},m_3,J_3) \\ {\dot{Q}}&= (0, \dot{q_1}, 0, \dot{q_2}, v_{2_{q_1}}, \dot{\psi _1}, v_{2_{q_2}}, \dot{\psi _2}, {\dot{y}}, {\dot{\varphi }})\\ n&= (1,1,1,1,3,3,3,3,3,3) \end{aligned}$$

To obtain the Euler–Lagrange equation, *L* was derived with respect to *q* and ${\dot{q}}$. In most dynamic systems, end-effector outputs, such as *y*, are calculated with the input degree of freedom, such as *q*. However, in this system, the relationship between *y* and *q* is a sixth-order equation, which cannot be directly expressed. Therefore, the differential terms $\frac{\partial L}{\partial {\dot{q}}} , \frac{\partial L}{\partial q}$ cannot be differentiated directly. Because *L* is described by *q*, *y*, using methods similar to those used in Eq. (16), we obtain $\frac{\partial y}{\partial q}$ for describing the terms $\frac{\partial L}{\partial {\dot{q}}}$ and $\frac{\partial L}{\partial {\dot{q}}}$.

The dynamic equation of the mechanism can be written as

23 $$\begin{aligned} D(q)\ddot{q} + C(q,{\dot{q}}){\dot{q}} = \tau \end{aligned}$$

where ${\dot{q}}$ and $\tau$ are the angular velocity and torque of the motor, respectively. The velocity and torque are the two factors that are given and constrained by the specification of the transformation motor. Using Eq. (23), it is possible to obtain the output linkage acceleration for a given motor at a given posture created by the mechanism.

## Optimization of linkage lengths

### Design variables for optimization

The goal of the transformable wheel is to climb steps higher than its radius in a secure manner. Ideally, a transformable wheel should be able to climb steps higher than its radius. The step height that a transformable wheel can climb is determined by the wheel radius, the transformation radius, and the transformation angle^20^. However, to obtain the static performance achieved by the transformation, it is crucial to ensure the transformation process, which is the dynamics of the transformation process. Therefore, for a given static performance, that is, a given radius and targeted workspace of the wheel, the output force must be maximized to guarantee the transformation of the mechanism to its shape.

**Figure 5:**
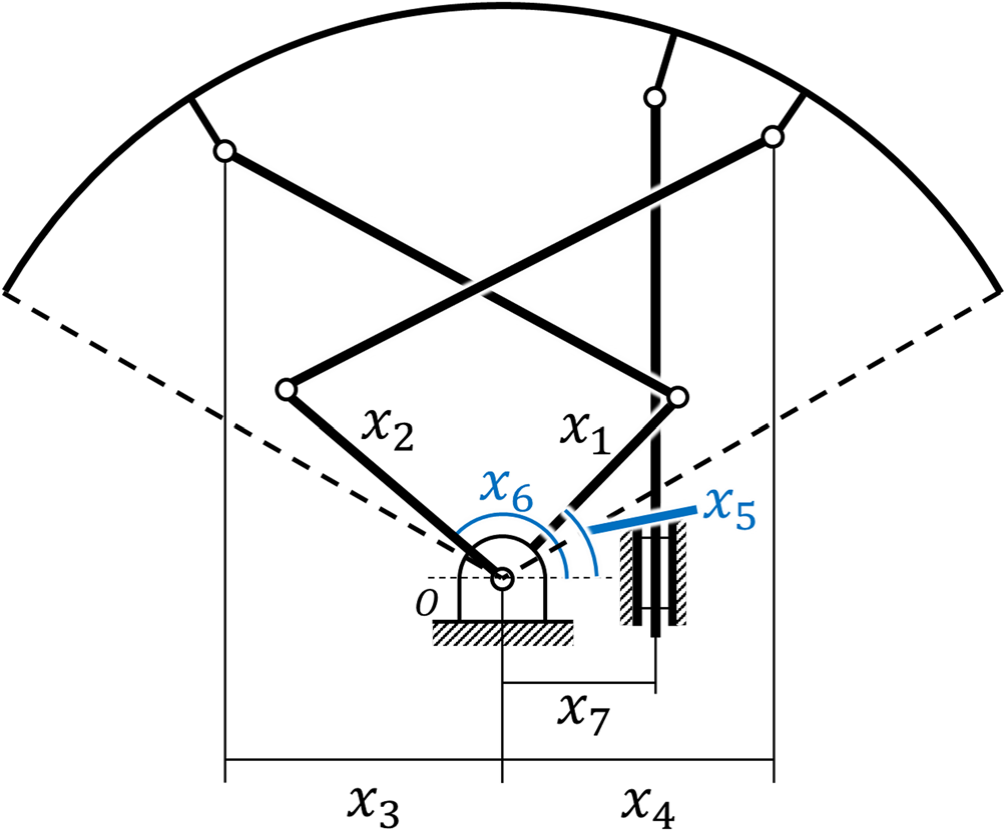
Design variables based on the non-transformed posture of the mechanism. The variables are restricted to the size of the motor armature and the fixed radius of the wheel.

Assuming that the wheel is in a full circle when closed, the design variables of the wheel can be defined, as shown in Fig. 5. The linkage lengths and angles define the required initial shape of the lobe and can then be kinematically calculated to determine the workspace of the defined mechanism. The radius of the wheel is 64.6 mm, excluding 17 mm of lobe thickness and bracket lengths from the prior wheel design in^20^, which targets a step height of 100 mm. The linear constraints on the different variables are listed in Table 2 and are restricted by the design of the motor armature and linkage. The distance from the center of the wheel to the LM guide *k* should not interfere with the other LM guides connected to the other two lobes.

24 $$\begin{aligned} x = [x_1\ x_2\ x_3\ x_4\ x_5\ x_6\ x_7] \end{aligned}$$

**Table 2:** Lower and upper boundaries on *x* for optimization. Constraints were set within the limits of the motor armature. The wheel’s radius was 64.6 mm and the workspace of each configuration had an offset of 5% with respect to the midpoint.

Variable	Lower bound	Upper bound
$x_1$	45 mm	60 mm
$x_2$	45 mm	60 mm
$x_3$	$x_7$	45.9452 mm
$x_4$	− 45.9452 mm	$x_7$
$x_5$	$30^\circ$	$60^\circ$
$x_6$	$120^\circ$	$150^\circ$
$x_7$	7 mm	55.9452 mm

### Optimization

The stability of the climbing mechanism is an important issue because the mechanism is targeted for using in robots or vehicles with delivery purposes. For measuring stability of a mobile robot or vehicle, mean value of acceleration perpendicular to the driving direction is suggested^25,26^. To ensure and increase stability, the output acceleration performance of the transformable wheel given by the two input motors is required to be at best. Specifically, the acceleration or force exerted on the rim from motor inputs must be maximum. However, the preceding research focuses on the functionality of the robot, which is the maximum height of the step that can be climbed. To maximize the output acceleration of the transformable wheel mechanism while maintaining the transformation functionality, optimization of the linkage structures is required.

An objective function was defined for optimization. The output force performance of the transformation mechanism was considered as the force exerted on the lobe from the center motor. Based on the results of the dynamic analysis, the acceleration of the lobe created by the given input torque and motor speed was evaluated. For a design condition given by *x*, the system’s performance varied throughout the available workspace, where the workspace also changed with the conditions of *x*. The workspace for each configuration was set from the original position of $x_5$ and $x_6$ to a 5% offset relative to the midpoint to avoid singularity of the mechanism.

**Figure 6:**
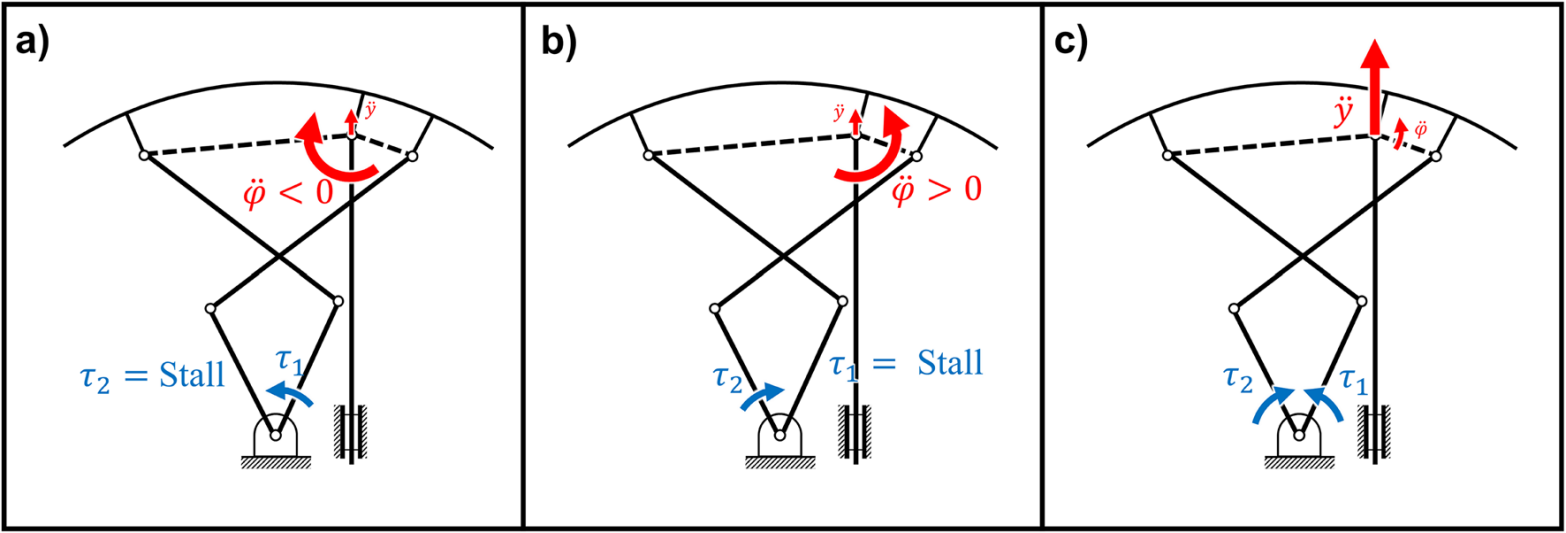
Three manipulator directions for the output of the mechanism. The input torque and speed are equal to those listed in the motor specifications. The dominant output is highlighted by wider red arrows.

**Figure 7:**
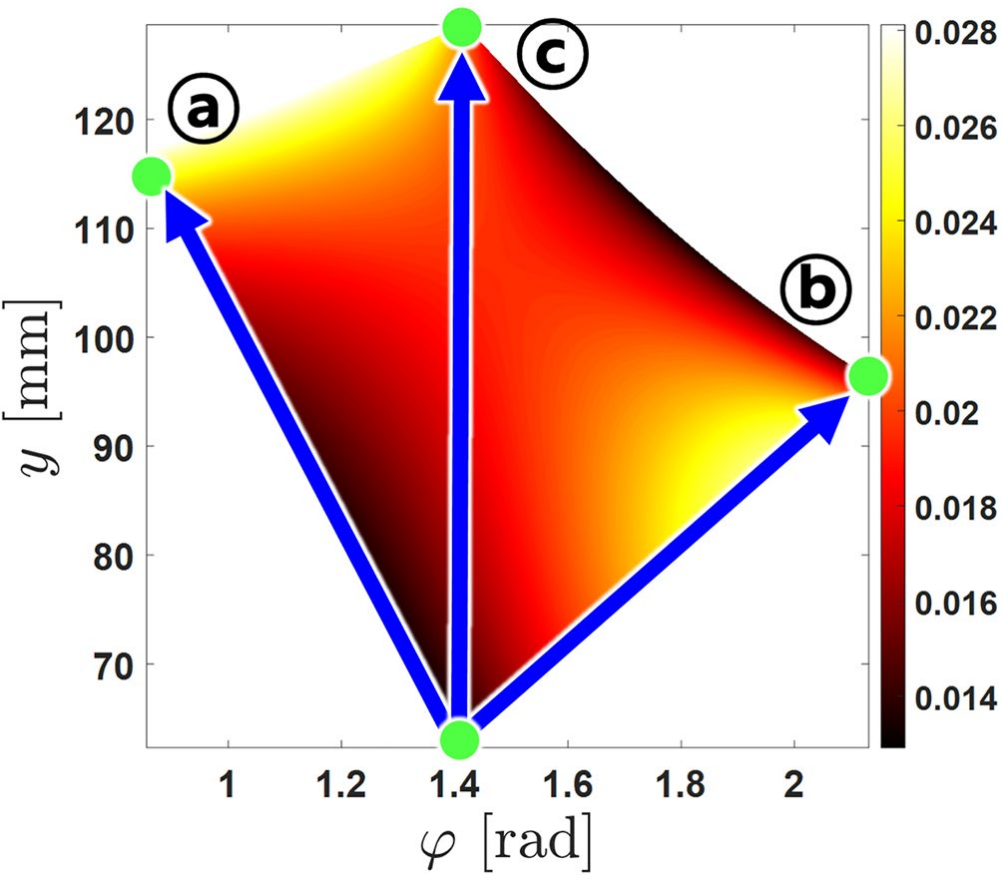
Three directions of the dominant output degree of freedom in example of the manipulability graph for the existing transformable wheel model. A control method for this mechanism is yet to be established, therefore manipulation is simple straight line through the workspace.

Three possible manipulator directions can be chosen for the operation of the transformable wheel, as shown in Figs.  6 and 7. Since there is no control method for this mechanism to follow a certain direction in the workspace for an optimized route, only the simplest manipulator routes were chosen to achieve the transformation. Directions (a), (b), and (c) are the degrees of freedom related to the output performance of the mechanism, with their own dominant output postures. To normalize the optimization, the mean performance in each direction for the given condition *x* was considered for obtaining the optimized performance. The mean performance value for each direction was used for normalizing the performance score for any given condition *x*. The normalized scores were then combined to obtain the performance score of *x*. However, there are transitional and rotational degrees of freedom $\ddot{y}(q),\ddot{\varphi }(q)$ to the lobe by the two motor inputs, both of which are required as objective functions for the output force performance. To simplify the process, the directions for each direction were scored with their dominant degrees of freedom. Summing the normalized scores eliminated dimensional differences between the linear and angular accelerations, making it possible to score both degrees of freedom.

All optimization processes were performed using the genetic algorithm method in MATLAB. The parameters for the GA are indicated in Table 3. The objective function was formulated as follows:

**Table 3:** The parameters and functions used in MATLAB genetic algorithm function.

GA parameters	
Population type	Double vector
Population size	100
Fitness scaling function	Rank
Selection function	Stochunif
Mutation function	Adapt feasible
Crossover function	Scattered
Crossover fraction	0.7
Migration interval	20
Migration fraction	0.1

25 $$\begin{aligned} \begin{array}{c} \min \\ x \end{array} f(x) = \frac{-\ddot{\varphi _1}(x)}{g_1(x_{\ddot{\varphi _1}})}+\frac{-\ddot{\varphi _2}(x)}{g_2(x_{\ddot{\varphi }_2})}+\frac{-\ddot{y}(x)}{g_3(x_{\ddot{y}})} \end{aligned}$$

where

26 $$\begin{aligned} \begin{aligned} \begin{array}{c} \max \\ x_{\ddot{\varphi }_1} \end{array} g_1(x_{\ddot{\varphi }_1})&= mean(|\ddot{\varphi _1}(x)|) \text {(workspace between initial to }x_5\text { limit)} \\ \begin{array}{c} \max \\ x_{\ddot{\varphi }_2} \end{array} g_2(x_{\ddot{\varphi }_2})&= mean(|\ddot{\varphi _2}(x)|) \text {(workspace between initial to }x_6\text { limit)} \\ \begin{array}{c} \max \\ x_{\ddot{y}} \end{array} g_3(x_{\ddot{y}})&= mean(|\ddot{y}(x)|) \text {(workspace between initial to }x_5 , x_6\text { limit)} \end{aligned} \end{aligned}$$

For each objective function in Eq. (26), there is an associated workspace for each optimization. For example, for $g_1(x_{\ddot{\varphi }_1})$, the workspace is configured from the initial position of $q_1$ which is $x_5$, to the limit of the 5% offset from the midpoint of initial input positions $x_5,x_6$. Other input degree of freedom $q_2$ is fixed for this optimization, therefore transformation torque is applied only in parallel to the changing input $q_1$. The output of this function is the mean value of the acceleration of the dominant output degree of freedom, $\ddot{\varphi } <0$. This workspace leads to the maximum angle displacement of the transforable wheel, which is the limit configuration for climbing a step. Therefore, the optimization leads to the best set of design variables *x* for operating the wheel lobe in the $\ddot{\varphi } <0$ direction. Similarly, for the other objective functions $g_2(x_{\ddot{\varphi }_2}),g_3(x_{\ddot{\varphi }_2})$, their optimization leads to the best set of *x* for operating in the respective targeted output.

## Results and analysis

### Reference optimization results

**Figure 8:**
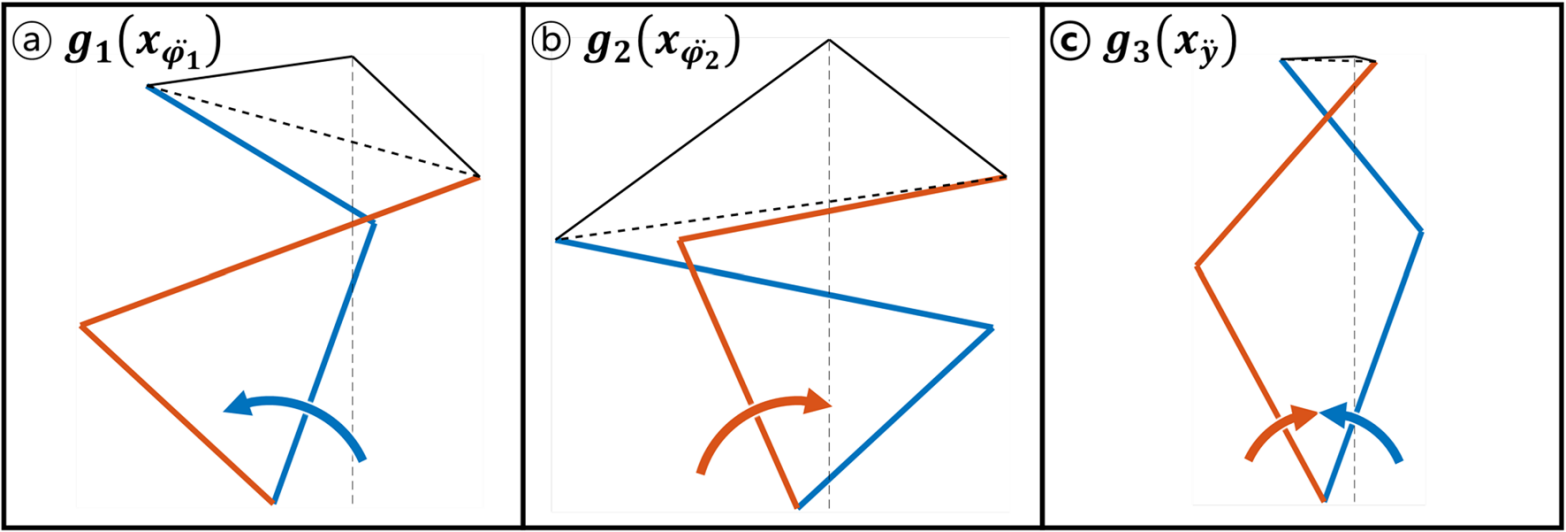
The visualization of the three optimized sets for the dominant degree of freedom. The design variables are mostly at the boundaries due to the limitations of the motor design.

Three separate optimization is first taken. For each directions (a), (b) and (c), their respective dominant degree of freedom is used as the objective function for the optimizations. Table 4 and Fig. 8 each represent the values of the optimized sets and their initial positions visualized for one part of the lobe out of the three. From the asymmetry cause by the location of the LM guide, the results showed unbalanced linkage postures for each optimum. Most of the design variables end up in the boundary conditions, which was expected due to the tight constraint from the design of the two motors.

**Table 4:** The three optimized sets for each degree of freedom. Each optimized value is used for the normalization of performance score for the optimization.

Objective function	Optimized design variables							Optimized value
	$x_1$ [mm]	$x_2$ [mm]	$x_3$ [mm]	$x_4$ [mm]	$x_5$ [rad]	$x_6$ [rad]	$x_7$ [mm]	
$\ddot{y}$	45.0023	45.0041	− 10.0001	11.9459	1.0471	2.2842	7.0803	631.0245 [mm/s$^2$]
$\ddot{\varphi }>0$	49.5995	45.0001	− 53.1179	45.9451	0.5236	2.1854	7.0001	7.1142 [rad/s$^2$]
$\ddot{\varphi }<0$	45.0000	49.6000	− 28.2679	45.9452	1.0472	2.6180	17.5851	− 8.7923 [rad/s$^2$]

For the three optimized values, the values are in theory, the best performance possible for a linkage configuration can make. By dividing each best values and adding the normalized values together, a performance score objective function that is always smaller than 3 was defined. Each normalized score has no weight whatsoever, since every score is equally significant of operating the transformation.

### Optimization results and analysis

Based on the three reference optimizations, the optimization score for maximizing the performance in the targeted output was established. Table 5 and Fig. 8 shows the optimized set of linkages based on the optimization score. As described in Table 5, the scores of acceleration in the direction of $\ddot{y}, \ddot{\varphi }>0, \ddot{\varphi }<0$ is found compared with the initial condition to the optimized condition. Torque output score in the $\ddot{y}$ direction increased from 0.4647 to 0.9433 which is about 103% increase. For torque transmission in the $\ddot{\varphi }>0$ direction increased in 166%, score from 0.3033 to 0.8063. Torque transmission in the $\ddot{\varphi }<0$ direction has the least affect, where the score increased from 0.4668 to 0.5006, resulting in 7.2% increase. The combined score of the three degrees of freedom increased from 1.2348 to 2.2501 which is 82% increase in torque output performance.

**Table 5:** Optimal design variables whose objective function is scored by the normalization from other optimums. Above is the normalized score for the initial design. All three performance was improved compared to the original model. Below is the optimized design variables optimized without $x_1$, $x_2$ limits. All objective function is normalized by the bounded optimal design parameters, therefore the sum of the score is possible to be above 3.

Objective function	Optimized design variables							Scores			
	$x_1$	$x_2$	$x_3$	$x_4$	$x_5$	$x_6$	$x_7$	$\ddot{y}$	$\ddot{\varphi }>0$	$\varphi <0$	sum
f(x)(inital)	55	55	− 35	35	0.7854	2.3562	17	0.4647	0.3033	0.4668	1.2348
f(x)	45.4577	45.2689	− 45.2928	45.6776	1.0056	2.5904	7.4438	0.9433	0.8063	0.5006	2.2501
f(x)(unbound)	15.8692	16.7731	− 13.5388	33.5159	0.7611	2.2768	7.1765	1.8326	2.0853	2.4712	6.3891

**Figure 9:**
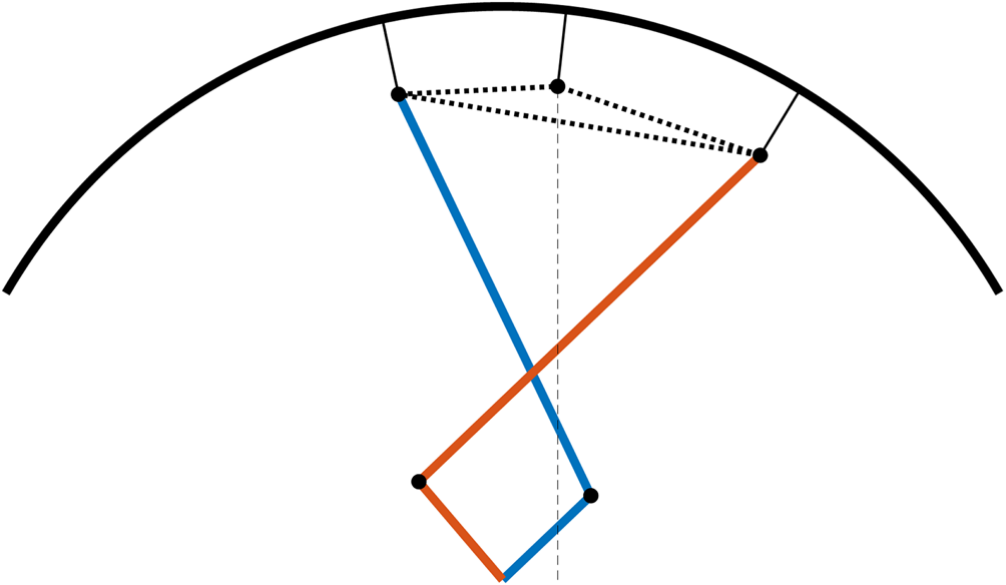
Visualization of the possible ideal form of the optimized set. This shape is impossible to manufacture due to the restrictions of the motor armature, however the input linkages can be concluded to be minimum to maximize performance of the 2-DOF wheel.

**Figure 10:**
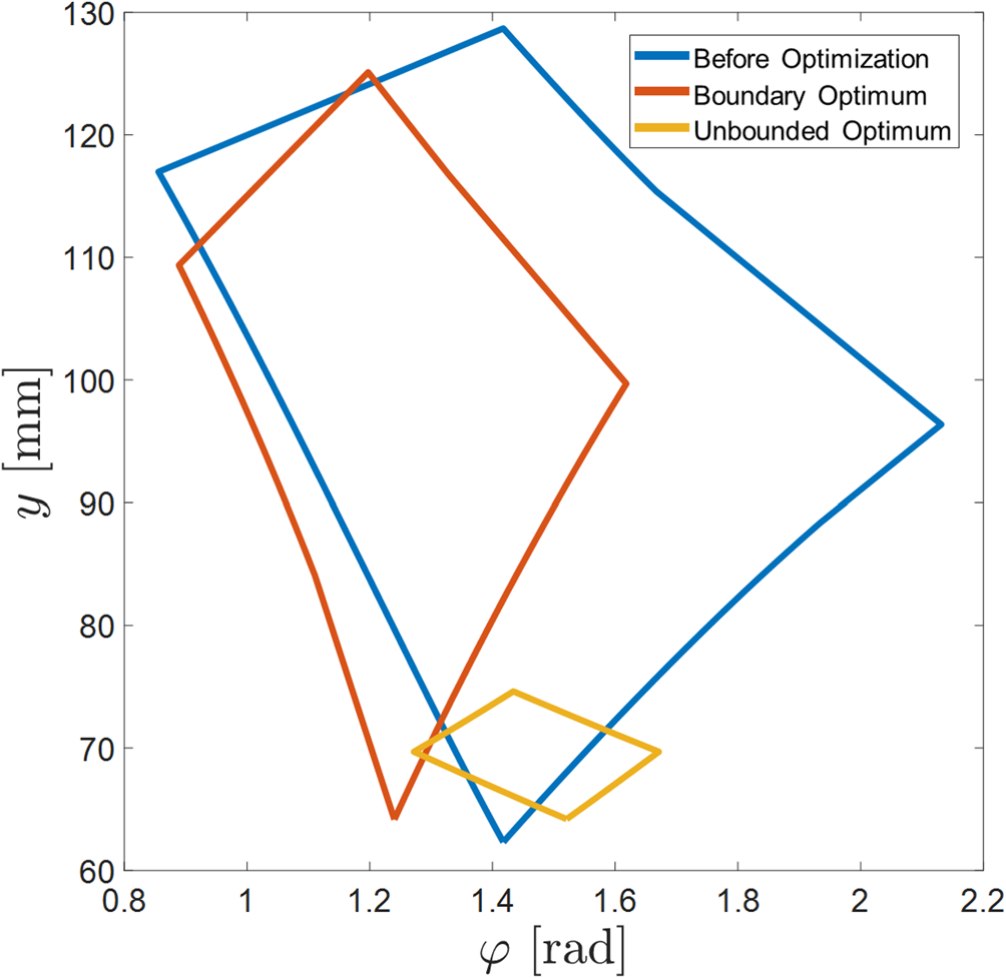
Workspaces of design variables before and after optimization. The area of the workspace is smallest with unbounded optimal design, therefore unsuitable for the mechanism.

The score showed that most of the design variables end near boundary condition, indicating that the optimum value is not within the selected boundary. Figure 9 shows the assumed optimum if the boundary conditions of the input linkages $x_1$ and $x_2$ were not restricted to the structure of the motor, with only physical constraints within the wheel radius as shown in Table 5. The score of the unbounded variable set is based on the restricted boundary conditions, so the sum of the scores were larger than 3. This seem the shorter the input linkages are, the better torque performance it has. This is true if seen dynamically, however as shown in Fig. 10, the workspace for the unbounded variable set is incredibly small compared to the reference design, due to the short input linkage lengths. Not only this ideal set is impossible for the motor to be made physically, but also the main purpose of the design to climb higher steps is discouraged. This can be concluded that the shorter input linkage variables $x_1$ and $x_2$ must be short as possible, but long enough to maintain the transformable mechanism’s capability to climb higher steps. Also, the length between the center of the wheel to the location of the LM guide is also crucial for the design. This asymmetrical design is unavoidable due to the installation of the LM guides not being able to overlap each other, therefore the guides must be offset from the center of the wheel. However it can be concluded that the location must be closest to the center for the increased performance of the wheel. The locations of the brackets on the rim, of $x_3$ and $x_4$ is dependent on the lengths of other design variables, as well as the initial input angles of $x_5$ and $x_6$ which can be determined by optimization.

**Figure 11:**
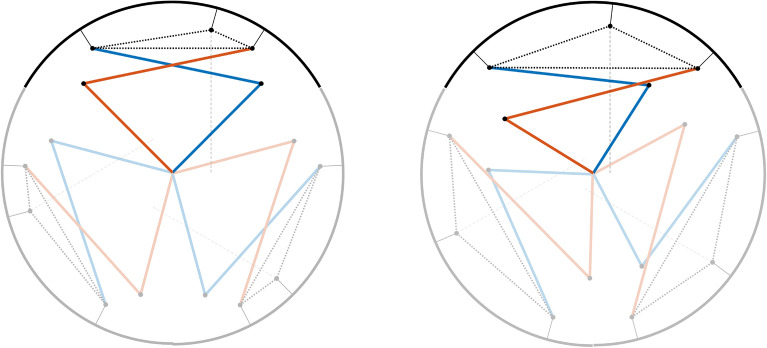
(Right)The schematic of the full wheel with one part of the lobe highlighted. (Left)The The proposed optimum linkage design set visualized. The shape of the wheel in full assembly can be seen with this diagram. There is no interference with other parts of the wheel, making proving this set to be plausible to manufacture.

As shown in Fig. 10, the workspace of the initial design is the larger than any other design sets. For the initial design of the transformable wheel mechanism, to increase the possibility of climbing a step with limited workspace, the linkages were chosen to be as long as possible. which guaranteed the wheel to climb steps higher than 100 mm. On the other hand, the optimized design sets have smaller workspace region compared to the initial design. The linkages in the unbounded optimal set are too short for an effective transformation to occur. Therefore, this can be expected that even if the structure of the motor is improved for the linkage designs to have longer constraints and generate more performance than the longer counterparts, some amount of linkage length is required to ensure the general purpose of the wheel. However, it can also be seen that even decent performance improvement can also lead to minimizing the workspace region. This trade-off relationship must be balanced to bring maximum performance for maximum functionality. A example of this balanced design is the boundary optimum design shown in Fig. 11. This design also has smaller workspace compared to the initial design, consequently having smaller climbable step size about 80 mm. Since the initial design is able to climb steps about 100 mm, it can be said that the main performance of the wheel is slightly lost. However, as shown in Table 5, the optimized set showed better performance of delivering torque to the output degrees of freedom.

## Conclusion

The kinematics and dynamics of the proposed 2-DOF mechanism were studied in the present work. The kinematics and dynamics were determined analytically, unlike in prior studies. The transformable wheel was optimized based on the mechanism’s dynamics. Within the targeted radius of the wheel, the linkage architecture was optimized, for maximizing step-climbing stability. To maximize this stability, the output force performance of the mechanism was optimized, to ensure that the acceleration to maintain stability is maximized. The optimization was performed using the genetic algorithm in MATLAB. The result presented on this research is yet to be validated with experiments because the physical model of the mechanism is currently not available for force analysis. The validation of the dynamics and optimization with force sensing capable platform is possible work in the future. The results of this work can be used for building larger-radius transformable wheel mechanisms for climbing higher steps with assured transformation performance, and therefore, with higher stability. The dynamics analyzed in this study are also available for the adaptation of control theories of transformable wheel mechanisms. The most foreseeable control is the compliance control of a transformable wheel, which targets the wheel to reduce the impact while driving in a transformed state.

## Data Availability

Due to space limitation, this paper only shows data results processed from raw data using the definition of the introduced dynamic analysis. The raw data generated during and/or analyzed during the current study are available from the corresponding author on reasonable request.
